# Electrical Study of Trapped Charges in Copper-Doped Zinc Oxide Films by Scanning Probe Microscopy for Nonvolatile Memory Applications

**DOI:** 10.1371/journal.pone.0171050

**Published:** 2017-01-30

**Authors:** Ting Su, Haifeng Zhang

**Affiliations:** 1 State Key Laboratory of Surface Physics and Department of Physics, Fudan University, Shanghai, China; 2 Department of Physics, Jiamusi University, Jiamusi, China; Institute of Materials Science, GERMANY

## Abstract

Charge trapping properties of electrons and holes in copper-doped zinc oxide (ZnO:Cu) films have been studied by scanning probe microscopy. We investigated the surface potential dependence on the voltage and duration applied to the copper-doped ZnO films by Kelvin probe force microscopy. It is found that the Fermi Level of the 8 at.% Cu-doped ZnO films shifted by 0.53 eV comparing to undoped ZnO films. This shift indicates significant change in the electronic structure and energy balance in Cu-doped ZnO films. The Fermi Level (work function) of zinc oxide films can be tuned by Cu doping, which are important for developing this functional material. In addition, Kelvin probe force microscopy measurements demonstrate that the nature of contact at Pt-coated tip/ZnO:Cu interface is changed from Schottky contact to Ohmic contact by increasing sufficient amount of Cu ions. The charge trapping property of the ZnO films enhance greatly by Cu doping (~10 at.%). The improved stable bipolar charge trapping properties indicate that copper-doped ZnO films are promising for nonvolatile memory applications.

## Introduction

Ferroelectric thin films have attracted much attention due to their potential application of nonvolatile random-access-memory devices [1]. Among potential candidates, copper-doped ZnO is one of the most promising because its excellent versatility in electrical properties [2,3]. Mishra *et al*. [4,5] successfully reported direct growth of freestanding ZnO tetrapod networks for multifunctional applications. Tiwari *et al*. [6] reported the observation of room temperature ferromagnetism in Cu-doped ZnO films, which are very important findings. The electrical property of the ZnO film is dominated by doped atom incorporated during production and growth processes. An efficient way of improving the properties of ZnO films is the addition of certain dopants. Kumar *et al*. [7,8] reported tailoring of extrinsic dopants provide interesting enhancement in electronic transport properties of ZnO films. It is well known that copper atoms in zinc oxide are electron traps. The interesting features of ZnO:Cu have inspired us to explore its electrical properties for potential charge storage application. Understanding the charge transport mechanism in copper-doped ZnO film is important and intriguing for memory applications. Little has been reported on the charge trapping property of Copper-doped ZnO films, which governs essential physics. Therefore, further studies are still needed to discover optimum Cu doping concentration in ZnO films for better charge trapping property.

In this study, We present the charge storage in ZnO:Cu films and use Kelvin probe force microscopy (KPFM) to demonstrate stable bipolar charge features. In addition, copper doping results in significant change in the Fermi level of the zinc oxide film. The KPFM measurement proves that increasing sufficient amount of Cu ions (~ 10 at.%) leads to the ohmic contact between the Pt-coated tip and ZnO:Cu films. Therefore, a large amount of charge was stored in the 10 at.% Cu-doped ZnO film. It suggests that the Cu-doped ZnO films are promising candidates for high-density charge storage application.

Kelvin probe force microscopy has proven to be an effective method for characterizing ferroelectric materials in nanoscale. KPFM is commonly believed to acquire a wide knowledge of electronic properties of functional material and interfaces [9–11]. KPFM has been widely used to provide the mapping of surface and interface with resolution ranging from hundreds of micrometers to the nanometer scale [10]. The Kelvin probe force microscopy is developed as a powerful technique to measure the contact potential difference (CPD) [9,12]. Due to the difference in the work functions between the sample (*ϕ*_*sample*_) and the conductive tip (*ϕ*_*tip*_), the contact potential difference (*V*_*CPD*_) can be defined as [12]

$$V_{CPD}=\frac{\phi_{tip}-\phi_{sample}}{q}$$ (1)

Moreover, KPFM provides quantitative information on local potential related to the presence of intrinsic surface states and the polarization [13,14]. Further investigations under cleaner surface conditions as well as measurements concerning changes of the contact potential difference due to changes in dopant concentration of semiconductors will provide new and additional information about sample properties [15]. Therefore, this work investigated the contact potential difference of the copper-doped ZnO films using the KPFM at ambient condition. The paper is organized as follows. In the following section, we describe the sample preparation and Kelvin probe force microscopy method. Our results and discussion are presented in the third section. The summary of this paper is given in the fourth section.

## Methods

The ZnO films have been grown by using a variety of growth techniques, including pulsed laser deposition, [16,17] chemical vapor deposition and molecular beam epitaxy. Tiwari and Jin *et al*. [18,19] successfully produced high-quality zinc oxide thin films on silicon using pulsed laser deposition technique. In this study, the 240-nm-thick pure ZnO film (reference sample) and copper-doped ZnO films with varying Cu concentration ranging from 2 to 10 at.% (2, 8, 10 at.%) were grown at 600°C using pulsed laser deposition (PLD) on Si/SiO2/Ti/Pt and (001) quartz substrate. A KrF excimer laser operating at a wavelength of 248nm and an average energy density of 1.8 J/cm^2^ per pulse was used during the PLD.

**Fig 1:**
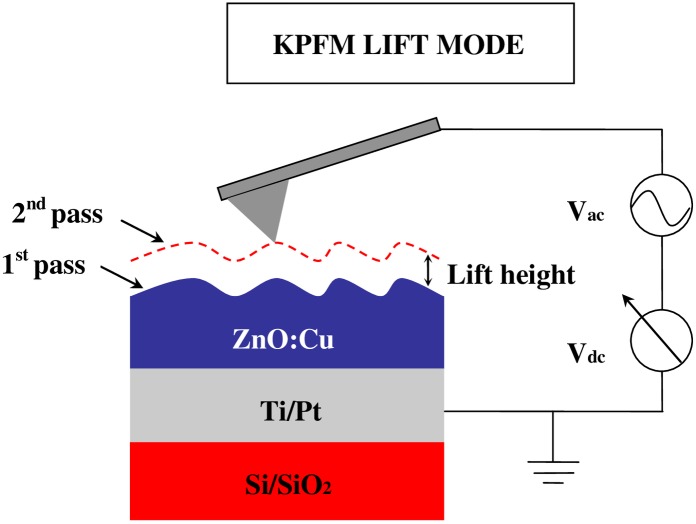
Sample structure and KPFM technique operating in lift mode. (Non-contact mode).

To investigate electronic properties of the copper-doped ZnO films, KPFM was performed on Asylum MFP-3D atomic force microscopy (AFM). Commercial software (Igor Pro 6.22A and Asylum Research, version 101010–2106) was used. Original surface potential of the undoped and copper-doped ZnO films were measured before charge injection. Moreover, AFM was performed in contact mode for applying electric field to investigate the charge trapping properties. In all experiments, we used conductive Pt-coated tip (Electri-Lever, Olympus, Japan, typical radius ~15nm) with resonant frequency ~70 kHz and spring constant ~2N/m. Fig 1 shows two-pass technique (lift mode), which is adopted in KPFM. The first pass is used to acquire topography of the surface. The second pass is used to measure CPD by lifting the tip up to a fixed distance above the surface [10]. KPFM measurements were carried out on two different areas of every sample. The experimental results are found to be consistent. The results are independent on the areas of measurements. KPFM scanning was operated in noncontact mode by applying 3V AC voltage to the tip. The tip was 40 nm above the surface to get good resolution, which is related to long-range electrostatic interactions. The voltage between sample and tip can be expressed by

$$V=V_{CPD}-V_{dc}+V_{ac}sin(\omega t)$$ (2)

Where *V*_*dc*_ is the DC offset potential applied to the tip, *ω* and *V*_*ac*_ are the frequency and amplitude of the applied AC voltage respectively.

KPFM measurements are governed by long-range electrostatic force. Therefore, the contribution of the probe and the cantilever is important [10,12]. The contact potential difference is determined to a large degree by the tip sharpness, cleanness and the conductive nature. It is reported that in some experiments, annealing and sputtering are adopted to clean the tip [12]. The electrostatic tip-sample interaction induces a mechanical oscillation of the cantilever [10]. If the system of the tip and the sample are modeled as a parallel plate capacitor, the force between the two plates can be expressed as

$$F=-\frac{1}{2}\frac{\partial C}{\partial z}V^{2}=-\frac{1}{2}\frac{\partial C}{\partial z}{\left[V_{CPD}-V_{dc}+V_{ac}sin\left(\omega t\right)\right]}^{2}$$ (3)

Where C is the capacitance between sample and tip, *z* is the tip-sample separation distance. Furthermore, feedback is applied to adjust the dc offset so that the *ω* component of the electrostatic force interaction is zero when the dc offset potential equals the contact potential.

## Results and Discussion

**Fig 2:**
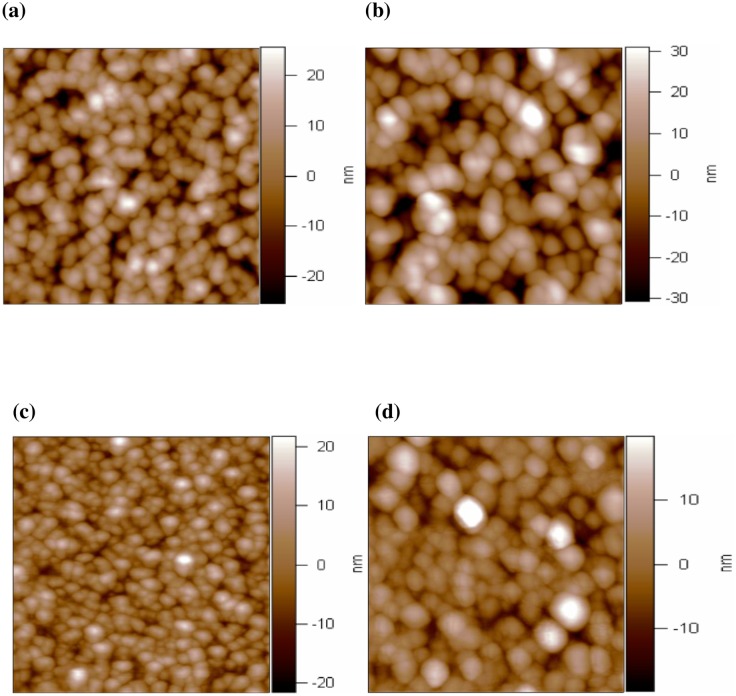
Topography images of (a) the undoped ZnO film, (b) 2% Cu-doped ZnO Film, (c) 8% Cu-doped ZnO Film, (d) 10% Cu-doped ZnO Film.

**Fig 3:**
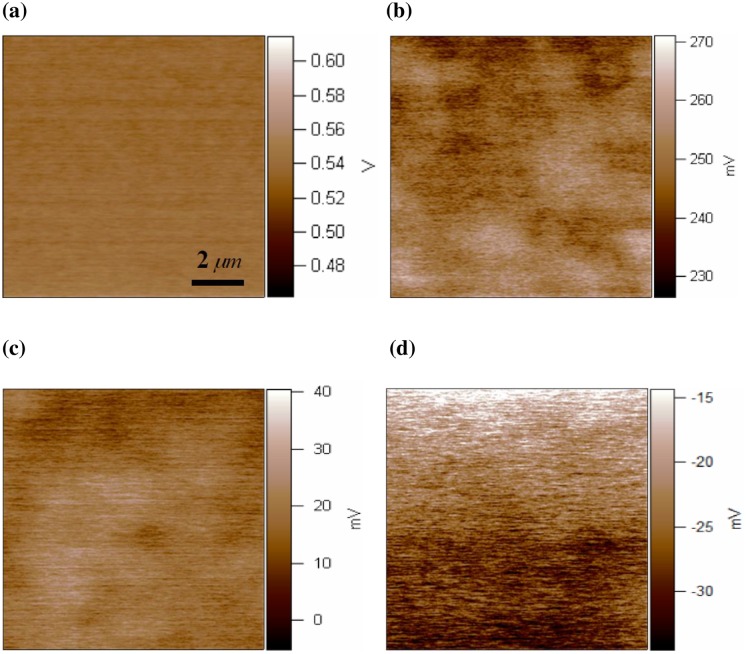
KPFM images of (a) the undoped ZnO film, (b) 2% Cu-doped ZnO Film, (c) 8% Cu-doped ZnO Film, (d) 10% Cu-doped ZnO Film.

Original surface potential of the undoped ZnO and copper-doped ZnO film were measured before charge injection. Fig 2 shows the topography images of undoped and copper-doped ZnO films. Fig 3(a) and 3(c) show surface potential images of the undoped ZnO and 8 at.% copper-doped ZnO film. The KPFM measurements show significant difference between the ZnO and ZnO:Cu film. The surface potential was 550mV and 18 mV for the ZnO and 8 at.% copper-doped ZnO film respectively. The difference in the Kelvin probe force microscopy data can be understood in terms of the doping-induced shift of the Fermi level. The position of the Fermi level can be changed with increasing doping level. The Fermi level [20], important in the physics of the semiconductor, also provides a good pictorial representation of the characteristics of the semiconductor material. Therefore, the position of the Fermi level reflects doping level of the copper-doped ZnO films.

Table 1 shows the CPD measured by Kelvin probe force microscopy before charge injection. The pure zinc oxide shows the highest CPD (550mV), compared to that of the 2 at.% copper-doped zinc oxide film (240mV) and 8 at.% copper-doped zinc oxide film (18mV). However, it is interesting to note that 10 at.% copper-doped zinc oxide film shows negative CPD (-25mV). This indicates the CPD values of the ZnO:Cu films decrease greatly by copper doping. Many models have been presented in order to explain the phenomenon observed at metal/semiconductor interface. To rationalize the observations, we introduce Mott-Schottky model, which is appropriate in our case. In the case of metal/semiconductor contact, it can be described with Mott-Schottky model [10]. KPFM experimental evidence (Fig 3a, 3b and 3c) demonstrates that the pure and light copper-doped ZnO film exhibit Schottky contact between the sample and Pt-coated tip. KPFM is commonly believed to study electronic properties of the surface and interface [10]. The CPD measured by KPFM is related to the differences in work functions between the sample and tip [10]. Metal-semiconductor interfaces form Schottky junctions due to the mismatch of their work functions and electron affinities [21]. For the Schottky contact on the undoped and light doped ZnO film, there exists potential barrier for electrons to cross from the sample to the tip.

**Table 1:** Measured Contact Potential Difference (CPD) by Kelvin Probe Force Microscopy (KPFM).

sample description	ZnO	ZnO:Cu 2%	ZnO:Cu 8%	ZnO:Cu 10%
V_CPD_ (mV)	550 ± 10	240 ± 6	18 ± 1	−25 ± 1
junction type	Schottky	Schottky	Schottky	ohmic

The measured CPD value of the 10 at.% copper-doped ZnO film is negative. Therefore, the KPFM measurements (Fig 3d) demonstrate the formation of Ohmic contact between Pt-coated tip and ZnO:Cu (~10 at.%). This shows significant contrast to the pure and light copper-doped ZnO film. These results indicate that the ZnO:Cu/tip interface is conversed from Schottky-type to Ohmic-type contact by increasing the amount of the copper ions. For the ohmic contact on a heavily doped ZnO, the barrier can be greatly reduced. In this case, the barrier becomes essentially transparent to the charge carriers. Therefore, it is easy for electrons to move between ZnO:Cu (~10 at.%) film and the tip. It is well known that the quality of metal-semiconductor contacts plays an important role in the performance of various semiconductor devices and integrated circuits. Good ohmic contacts [22,23] are essential for achieving excellent performance of a semiconductor device, while Schottky contacts [22,23] can be used for a wide variety of device applications.

**Fig 4:**
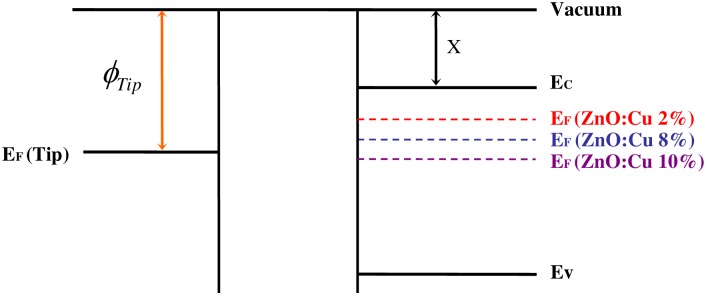
Schematic band diagram, showing the position of the Fermi level of different samples, where as EC = energy level of conduction band; EF = Fermi energy level; EV = energy level of valence band; *ϕ*_*tip*_ = work function of tip.

**Fig 5:**
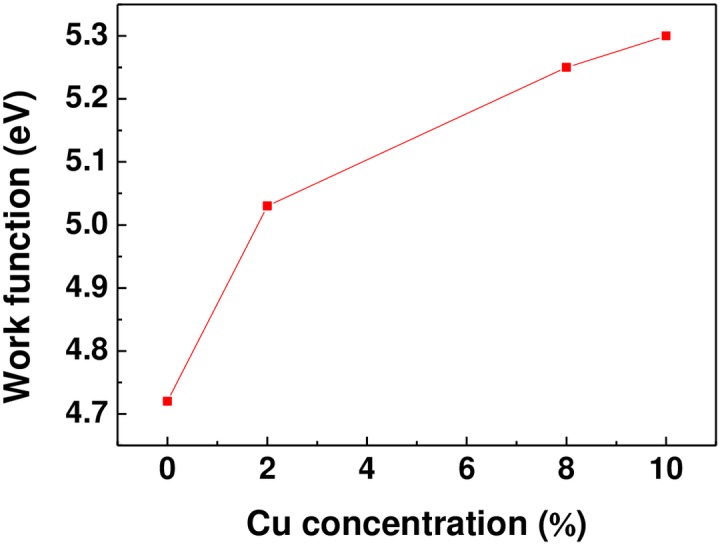
Work function of copper-doped ZnO film as a function of doping concentration.

The Fermi level position in semiconductors depends on the doping concentration [24,25]. ZnO is inherently n-type [24] semiconductor and has a wide bandgap of approximately 3.37 eV [2]. Fig 4 shows schematic band diagram of copper-doped ZnO films. The Fermi level of copper-doped zinc oxide films shift toward the valence band maximum by increasing the level of Cu doping. Moreover, from the KPFM data, the difference in the Fermi level of various materials can be found. Therefore, the Fermi level shift between the undoped ZnO film and 8 at.% copper-doped ZnO film is 0.53 eV, which is prominent compared with band gap. This suggests that the effect of copper doping on the electronic properties of the zinc oxide films results in the Fermi level change, which has a great potential in improving the charge trapping. In addition, we also observed the work function modification in copper-doped zinc oxide films revealed by Kelvin probe force microscopy. Fig 5 shows work function of copper-doped ZnO film as a function of doping concentration. Interestingly, Fermi level of the 10 at.% copper-doped ZnO film moves closer to the valence band, which results in the Schottky-Ohmic conversion at contact interface. Therefore, this intriguing observation inspired us to explore the charge trapping property of the 10 at.% Cu-doped ZnO film.

**Fig 6:**
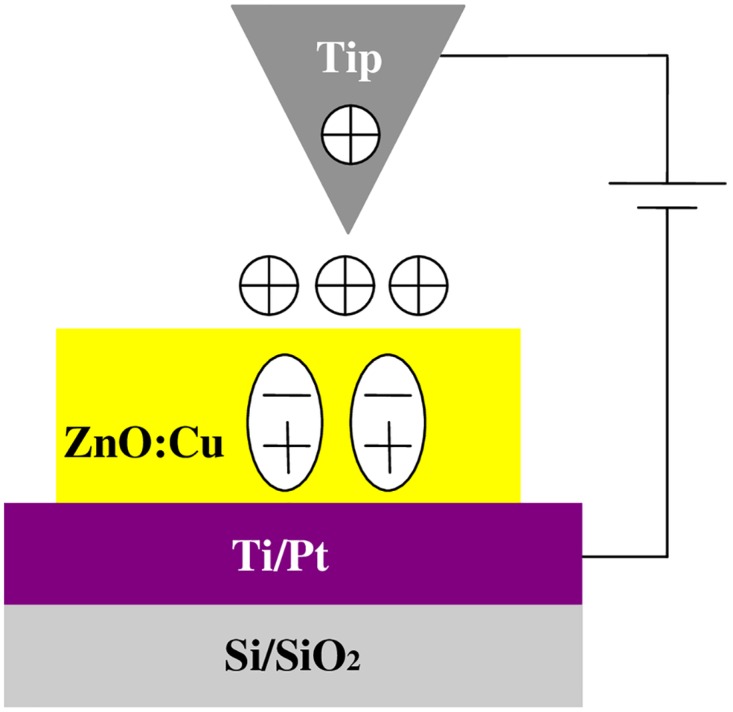
Sample structure and schematic of local poling of the copper-doped ZnO by the tip.

Fig 6 shows the schematic of local poling of the copper-doped ZnO by the tip. The efficiency of the injection process depends on the tip/sample contact [10]. When the copper doping concentration increases, the nature of contact at sample/tip interface is ohmic contact. Therefore, more charge carriers can be injected into copper-doped ZnO film due to the decreased barrier at the contact interface. During charging process, the conductive tip approached closely to the sample surface. Charge will be transferred to the surface and screen polarization charges of the dipoles when dc voltage is applied to copper-doped ZnO by the conductive tip. The surface potential of a polarized area is the sum of what the surface charge generates and the dipoles in the area contribute [1].

**Fig 7:**
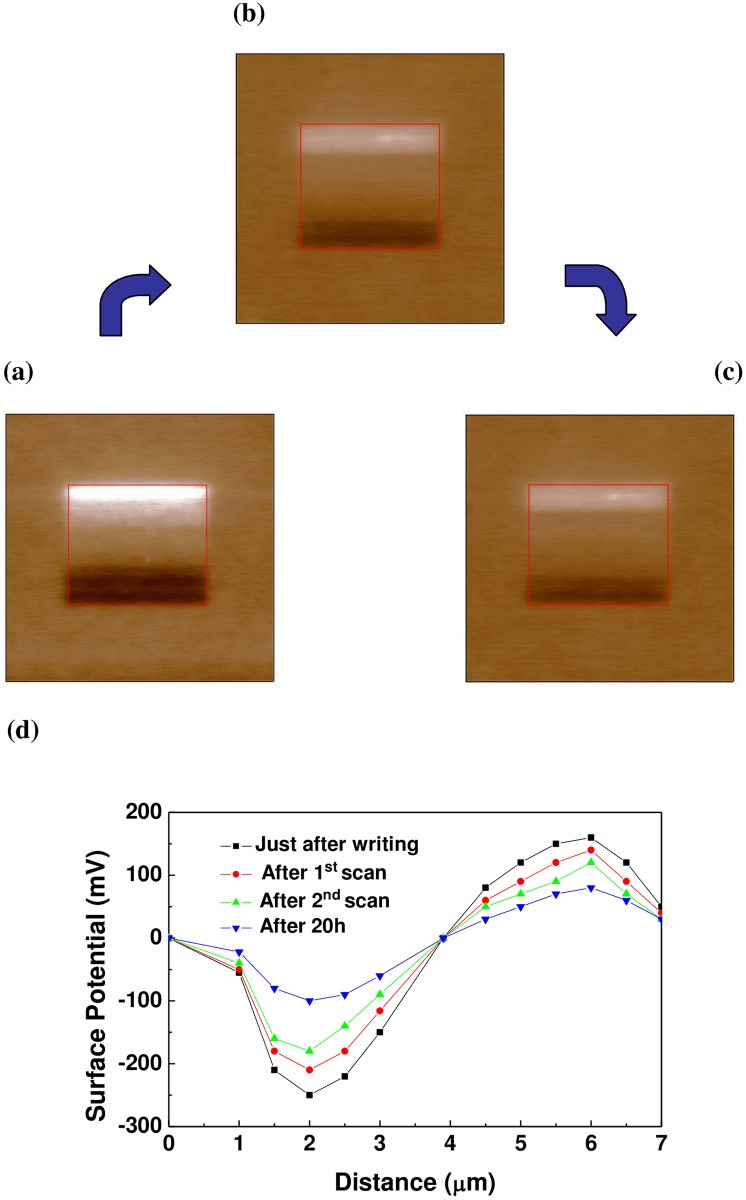
KPFM surface potential images of the 10% Cu-doped ZnO film after (a) bias application (b) the first grounded tip scan (c) the second grounded tip scan (d) KPFM data acquired from (a)-(c) and after 20h.

**Fig 8:**
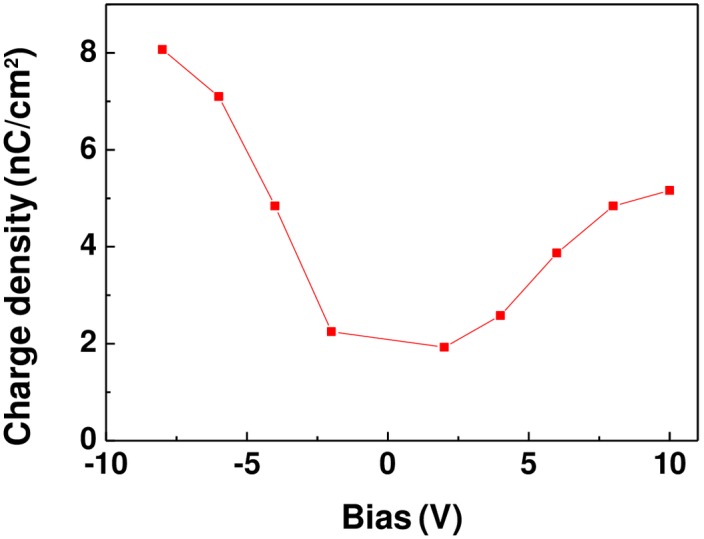
Charge density of copper-doped ZnO film as a function of applied bias.

To investigate the charge trapping property of the 10 at.% copper-doped ZnO film and surface potential change after a dc voltage bias, a series of dc biases, ranging from -8 to +10V with a step of 2V, was applied to the 10 at.% copper-doped ZnO film, as shown in Fig 7(a). Fig 7(a) shows the KPFM image obtained immediately after poling. The bright region represents positive potential and the dark represents negative potential. The applied bias dependence of the surface potential indicates that surface potential is determined by the compensation between screen charges and polarization. Furthermore, the discharging experiments have been performed to investigate the role of trapped electrons and holes. For discharging process, the stability of surface potential charge was studied by grounded tip scans, which procedure is similar to that of Kim et al [9]. Fig 7(b) and 7(c) show the KPFM images of the 10 at.% copper-doped ZnO film after the first and second scans by grounded tip. The surface charge was discharged by grounded tip scan [9]. However, there are still large amount of charge stored in the 10 at.% copper-doped ZnO film. Therefore, this indicates that the charge stored in the copper-doped ZnO film are mainly polarization and injection charge. From the KPFM data, it can calculate the surface charge density by parallel capacitor model [1]. The relative dielectric constant [26] of the ZnO film is 8.75. The remnant polarization [26] is 0.4*μC* / *cm*^2^. Fig 8 shows charge density of copper-doped ZnO film as a function of applied bias. The charge density of the copper-doped ZnO film was calculated to be 2*nC* / *cm*^2^–8*nC* / *cm*^2^. The overscreen ratio was calculated to be 1%-2%.

The stability of trapped charges in the 10 at.% copper-doped ZnO film was further studied by monitoring the time evolution of surface potential after biased tip engagement. The peak value of surface potential noticeably decays within 100 minutes after poling because the surface charge dispersed on the surface. Then the decay decreases after 200 minutes and the surface potential become stable. The CPD of the 10 at.% copper-doped ZnO film was measured at 20 h after poling, as shown in Fig 7(d). In this study, it is found that 40%-50% of initial charges are stored in the 10 at.% copper-doped ZnO films after 20 h, which are related to oxygen vacancy in zinc oxide. Oxygen vacancy may trap electrons and improve the charge trapping stability. Moreover, the copper dopants in ZnO act as deep acceptor to trap electrons [27]. The chemical state of Cu in ZnO can be present as Cu^+^ and Cu^2+^ ions substituting for Zn^2+^ ions [28]. Cu^2+^ is able to trap the electrons in its 3d orbital [29]. Oxygen vacancy and copper dopants are important for determining the electronic properties of the ZnO. The KPFM results accurately explain the electrical properties of the metal/semiconductor interface [10]. For the light doped ZnO film, the nature of contact at sample/tip interface is Schottky contact. However, as the copper doping concentration increases, for the 10 at.% copper-doped ZnO film, the nature of contact at sample/tip interface is ohmic contact. Therefore, a greater quantity of bipolar charge is stored in the 10 at.% copper-doped ZnO film. The improved stable bipolar surface charge features indicate that copper-doped ZnO films are suitable for applications in nonvolatile memories. Both copper dopants and oxygen vacancy improve the charge storage property, providing insight into physical understanding of charge transport in copper-doped ZnO for nonvolatile memory applications.

## Conclusions

In conclusion, Kelvin probe force microscopy is a powerful technique for measuring the nanometerscale surface potential and optimizing the design and performance of new devices based on semiconductor nanostructures. We have studied the charge trapping properties for both electron and hole in copper-doped zinc oxide films by KPFM. The KPFM measurements demonstrate that for ZnO:Cu (~10 at.%), copper doping results in important change in the Fermi level, which has potential to improve the charge trapping. In addition, increasing appropriate amount of Cu ions (~10 at.%) leads to the conversion from Schottky contact to Ohmic contact at Pt-coated tip/ZnO:Cu interface, which are proved by KPFM. Therefore, a greater amount of charge can be effectively trapped and stored in the films for a long time. The copper-doped ZnO films exhibit enhanced bipolar charge trapping properties and show great promise for nonvolatile memory applications.
