# Expression of Concern: Electrical Study of Trapped Charges in Copper-Doped Zinc Oxide Films by Scanning Probe Microscopy for Nonvolatile Memory Applications

**DOI:** 10.1371/journal.pone.0288213

**Published:** 2023-06-29

**Authors:** The PLOS ONE Editors

After publication of this article [1], concerns were raised that previously published work [2–4] that studied similar materials systems was not cited and discussed in article [1].

Additionally, contrary to the declaration in the Data Availability Statement, and in violation of PLOS’ Data Availability Policy, the original data files supporting the article’s results were not provided with the article. The authors did not provide the original raw data supporting results reported in this article [1] during follow-up discussions.

Upon follow up the *PLOS ONE* Editors were also unable to obtain information about where the experimental work was carried out.

Given that this article [1] does not comply with PLOS’ Data Availability Policy, the *PLOS ONE* Editors issue this Expression of Concern.
